# Ubiquitin-specific proteases are differentially expressed throughout the *Schistosoma mansoni* life cycle

**DOI:** 10.1186/s13071-015-0957-4

**Published:** 2015-06-26

**Authors:** Roberta V. Pereira, Matheus de S Gomes, Roenick P. Olmo, Daniel M. Souza, Fernanda J. Cabral, Liana K. Jannotti-Passos, Elio H. Baba, Andressa B. P. Andreolli, Vanderlei Rodrigues, William Castro-Borges, Renata Guerra-Sá

**Affiliations:** Núcleo de Pesquisas em Ciências Biológicas, Universidade Federal de Ouro Preto, Morro do Cruzeiro, Ouro Preto, MG Brasil; Instituto de Genética e Bioquímica, Universidade Federal de Uberlândia, Patos de Minas, MG Brasil; Departamento de Fisiologia, Instituto de Ciências Biomédicas, Butantã, SP Brasil; Centro de Pesquisas René Rachou, Fiocruz, Belo Horizonte, MG Brasil; Faculdade de Medicina de Ribeirão Preto, Universidade de São Paulo, São Paulo, Brasil; Departamento de Ciências Biológicas/Núcleo de Pesquisas em Ciências Biológicas - Instituto de Ciências Exatas e Biológicas - ICEB2, Universidade Federal de Ouro Preto, Sala 045, Campus Morro do Cruzeiro, 35400-000 Ouro Preto, MG Brasil

**Keywords:** Ubiquitination, Deubiquitination, Deubiquitinating enzymes (DUBs), Differential expression, Schistosome development

## Abstract

**Background:**

The ubiquitination process can be reversed by deubiquitinating enzymes (DUBs). These proteases are involved in ubiquitin processing, in the recovery of modified ubiquitin trapped in inactive forms, and in the recycling of ubiquitin monomers from polyubiquitinated chains. The diversity of DUB functions is illustrated by their number and variety of their catalytic domains with specific 3D architectures. DUBs can be divided into five subclasses: ubiquitin C-terminal hydrolases (UCHs), ubiquitin-specific proteases (USPs or UBPs), ovarian tumour proteases (OTUs), Machado-Joseph disease proteases (MJDs) and JAB1/MPN/Mov34 metalloenzymes (JAMMs).

**Methods:**

Considering the role that the ubiquitin-proteasome system has been shown to play during the development of *Schistosoma mansoni*, our main goal was to identify and characterize *Sm*USPs. Here, we showed the identification of putative ubiquitin-specific proteases using bioinformatic approaches. We also evaluated the gene expression profile of representative USP family members using qRT-PCR.

**Results:**

We reported 17 USP family members in *S. mansoni that present a conservation of UCH domains. Furthermore,* the putative *Sm*USP transcripts analysed were detected in all investigated stages, showing distinct expression during *S. mansoni* development. The *Sm*USPs exhibiting high expression profiles were *Sm*USP7, *Sm*USP8, *Sm*USP9x and *Sm*USP24.

**Conclusion:**

*S. mansoni* USPs showed changes in expression levels for different life cycle stages indicating their involvement in cellular processes required for *S. mansoni* development. These data will serve as a basis for future functional studies of USPs in this parasite.

**Electronic supplementary material:**

The online version of this article (doi:10.1186/s13071-015-0957-4) contains supplementary material, which is available to authorized users.

## Background

The covalent modification of proteins by the addition or removal of ubiquitin, a highly conserved protein comprising of 76 amino acids, changes the molecular function of the target protein and can therefore influence its interactions with other proteins. In turn, these interactions can regulate many biological processes, including DNA repair, cell-cycle control, endocytosis, transcription and protein degradation by the proteasome [1–4].

Deubiquitinating enzymes (DUBs) catalyse the removal of ubiquitin from ubiquitin-conjugated proteins and from its precursor proteins [5, 6]. DUBs can be divided into five subclasses, of which four are cysteine proteases and one comprises a group of metalloproteases. They are named as ubiquitin C-terminal hydrolases (UCHs), ubiquitin-specific proteases (USPs or UBPs), ovarian tumour proteases (OTUs), Machado-Joseph disease proteases (MJDs) and JAB1/MPN/Mov34 metalloenzymes (JAMMs) [7]. Of these, USPs represent the largest subclass with approximately 56 members in humans. The USP catalytic domain is highly divergent in size (295–850 residues). High sequence homology is mainly observed in two regions that surround the catalytic Cys and His residues: the so-called Cys Box domains, containing 19 amino acids, and the His Box domains, containing 60–90 amino acids [8–10].

As is the case for most cellular enzymes, the activity of DUBs can be controlled through multiple mechanisms. Several DUBs require assembly into large multimolecular complexes for full activation; this is exemplified by proteasomal DUBs. Other DUBs, including USP1, USP7, USP12 and USP46, are allosterically regulated by co-activator complexes or proteins, such as the WD40-repeat proteins, RAD50 and cullin-3 [7, 11]. Cross-talk between phosphorylation and ubiquitination is a significant aspect of intracellular signalling networks [12, 13]. Differential phosphorylation and dephosphorylation of some USPs can result in enhanced or reduced activity of these enzymes. Other post-translational modifications are emerging as modifiers of their activity, including ubiquitination and SUMOylation [7]. Furthermore, DUBs are prone to reversible inactivation caused by reactive oxygen species (ROS) [14].

Schistosomes are parasitic worms that require several coordinated morphological and biochemical changes that guarantee adaptation to various environments such as water and the internal milieu of their vertebrate and invertebrate hosts [15, 16]. Our group was the first to observe that the ubiquitin–proteasome system plays a crucial role in regulating cercariae to schistosomula transition in *Schistosoma mansoni* [17, 18]. Subsequent studies also revealed both differential expression of 20S proteasome subunits and specific patterns of ubiquitinated proteins during *S. mansoni* egg maturation, highlighting the importance of controlled protein turnover during embryo development [19].

Considering the aforementioned results, the main objective of this work was to identify the putative and non-annotated genes encoding ubiquitin-specific enzymes using bioinformatic approaches. For this we took advantage of the available *S. mansoni* sequences as 81 % of the parasite’s genome has been assigned to specific chromosomes [20]. Additionally, we evaluated the gene expression profile of 17 identified members of the USP family. These may be involved in important cellular processes during the life cycle of this parasite.

## Methods

### Ethics Statement

All experiments involving animals were authorized by the Ethics Committee for Animal Care of the Federal University of Ouro Preto (CEUA/UFOP protocol no. 2011/55). The experiments were performed in accordance with national and international regulations accepted for laboratory animal use and care. Mice (Balb/c strain, age 6 weeks, weight ~ 16–18 g) were kept under environmentally controlled conditions (temperature ~ 25 °C; humidity ~70 %) with free access to water and rodent diet.

### Parasites

The LE strain was maintained by routine passage through *Biomphalaria glabrata* snails and BALB/c mice. The infected snails were induced to shed cercariae under light exposure for 2 h, and the larvae were recovered by sedimentation on ice. Adult parasites were obtained by liver perfusion of mice after 50 days of infection. Livers of infected mice were macerated in phosphate buffer (Na_2_HPO_4_ 63 mM_,_ KH_2_PO_4_ 330 mM, pH 8.2) and trypsinised, and the homogenate was then incubated for 2.5 h at 37 °C in a water bath. Eggs were recovered in saline solution after sequential sieving of the liver homogenate through 360- and 180-μm meshes. Mechanically transformed schistosomula (MTS) were prepared as described by Harrop and Wilson (1993) [21], using a protocol that mimics skin-transformed *S. mansoni* schistosomula [22]. Briefly, cercariae were recovered and washed in RPMI 1640 medium (Invitrogen, Sao Paulo, Brazil) before vortexing at maximum speed for 90 s. The cercariae were immediately cultured in 169 medium for 3.5 h at 37 °C with 5 % CO_2_. Then, the recovered schistosomula were washed in RPMI 1640 until no tails were detected. For subsequent incubations, the parasites were maintained in M169 medium supplemented with 10 % FBS, penicillin/streptomycin at 100 μg/mL and 5 % Schneider's medium [23] at 37 °C in a 5 % CO_2_ incubator for 3.5, 24, 48 and 72 hours.

### Computational analysis of USPs

The putative *Sm*USPs were identified and selected by mining *S. mansoni* sequences in GeneDB database (version 5.0, available at http://www.genedb.org/genedb/smansoni/) using the basic local alignment search tool (BLAST) algorithm BLASTp and *Homo sapiens* reference USP proteins as queries [24]. Best Blastp hits showing cut-off values < e^−12^ were selected. Reference proteins from *Mus musculus*, *Rattus norvegicus*, *Drosophila melanogaster* and *Caenorhabditis elegans* were searched in the NCBI (National Center for Biotechnology Information) using BLASTp tool and non-redundant database to obtain a full set of putative orthologue USP proteins to compare with the *S. mansoni* putative proteins. Analyses of protein families, domains and active sites were performed using the PFAM (version 27.0, available at http://pfam.sanger.ac.uk) and Conserved Domains Database (CDD) (http://www.ncbi.nlm.nih.gov/cdd/) [25]. The entire protein sequences were used to perform multiple sequence alignments using CLUSTALX2 with the default settings (available at http://www.clustal.org/clustal2/) [26, 27]. A phylogenetic tree was inferred using the neighbour-joining method and the Jones-Taylor-Thornton model [28]. A bootstrap consensus tree inferred from 1,000 replicates was used to represent the evolutionary history of the taxa analysed. The molecular phylogenetic analyses were conducted using MEGA 5 software [29]. All positions containing gaps and missing data were eliminated from the dataset. Furthermore, the Kyoto Encyclopedia of Genes and Genomes (KEGG) database (available at http://www.genome.jp/kegg/) was used to search for orthologue proteins in *S. mansoni* compared with Eukaryote protein-coding genes generated from the GENES database in KEGG [30]. The protein domain logos were generated using WebLogo 2.8.2 at http://weblogo.berkeley.edu/logo.cgi [31].

### Expression analysis of identified USPs

Total RNA from cercariae, schistosomula, adult worms and eggs was obtained using a combination of TRIzol (GIBCO, Sao Paulo, Brazil) and chloroform extraction; it was then column-purified using the SV Total RNA Isolation System (Promega, Belo Horizonte, Brazil). The preparation was treated three times with RNase-free DNase I (1 unit each treatment), as described by the manufacturer (RQ1 DNase; Promega). The RNA was quantified using a spectrophotometer, and an aliquot containing 1 μg of total RNA was reverse transcribed using an oligodT primer from the ThermoScript RT-PCR System (Invitrogen) as described by the manufacturer. The efficiency of DNAse I treatment was evaluated by PCR amplification of a cDNA reaction mix lacking the ThermoScript enzyme. *S. mansoni-*specific primers were designed using the GeneRunner® program. Despite the fact that USPs have well-conserved sequences, primers were designed to less conserved regions. The sequence accession numbers and their primer pairs are shown in Additional file 1: Table S1. Reverse-transcribed cDNA samples were used as templates for PCR amplification using SYBR Green Master Mix UDG-ROX® (Invitrogen) and a 7300 Real-Time PCR System (Applied Biosystems, Rio de Janeiro, Brazil). *S. mansoni* EIF4E was used as an endogenous control (GeneDB ID: Smp_001500) (forward primer: 5’-TGTTCCAACCACGGTCTCG-3’, reverse primer: 5’-TCGCCTTCCAATGCTTAGG-3’) [32]. The efficiency of each pair of primers was evaluated according to the protocol developed by the Applied Biosystems application (the cDNA dilutions used were 1:4, 1:16, 1:64, 1:256 and 1:1024). The absence of non-specific products was confirmed by the presence of a single peak in the dissociation curves. For all investigated transcripts, three biological and technical replicates were performed, and their gene expression levels normalized using the EIF4E transcript as a reference according to the 2^−ΔCt^ method [33] using the Applied Biosystems 7300 software.

### Statistical analysis

Statistical analysis was performed using the GraphPad Prism software package, version 5.0 (Irvine, CA, USA). The normality of the data was established using one-way analysis of variance (ANOVA). Tukey post-tests were used to investigate significant differences in the expression of transcripts throughout the investigated stages. In all cases, the differences were considered significant when the *p* values were < 0.05.

## Results and Discussion

Here, we report 17 USP family members in *S. mansoni* and a particular emphasis was given to their structures and conserved domains. Phylogenetic analysis was conducted to understand how closely related these proteases are when compared to their orthologs. In addition, the *S. mansoni* orthologue proteins were compared throughout the parasite’s life cycle and their expression profile evaluated by qRT-PCR.

### Conservation of UCH domains in *S. mansoni* USPs

**Fig. 1:**
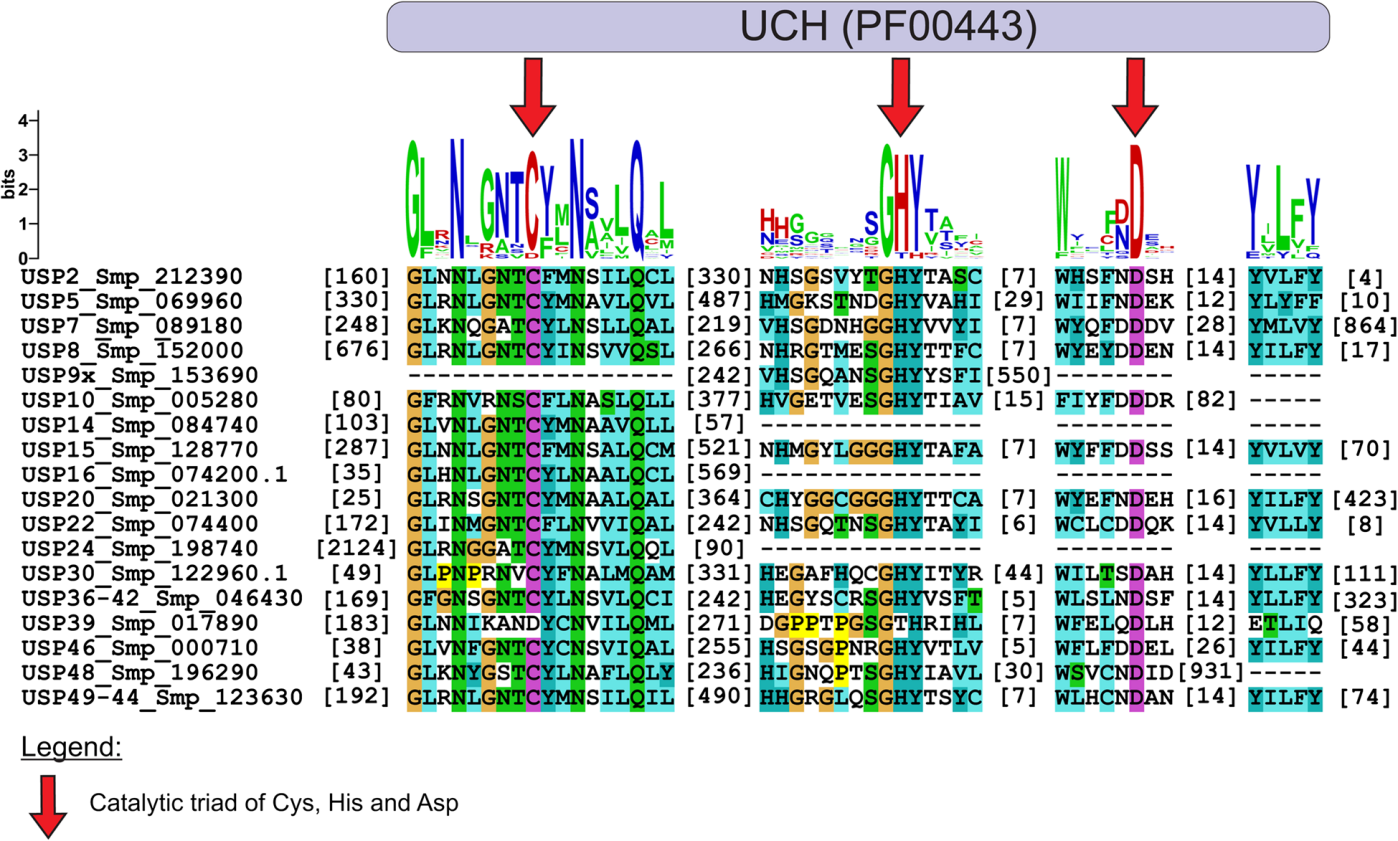
UCH domain alignment among *S. mansoni* USPs Eighteen USP genes with complete and incomplete UCH domain are shown. Arrows indicate conserved catalytic triad residues composed by cysteine, histidine and aspartic acid. Consensus logos are generated using WebLogo

Putative members of the USP family in *S. mansoni* were retrieved by mining the parasite databases. Our analyses revealed that *Sm*USPs are conserved at the amino acid level compared to their orthologues from diverse organisms, such as *D. melanogaster*, *C. elegans*, *H. sapiens*, *M. musculus* and *R. norvegicus*. Furthermore, by comparing the *S. mansoni* predicted genes and their related ESTs (Expressed Sequence Tag) from *S. japonicum* in NCBI, up to 54 % sequence similarity could be found (Additional file 1: Table S2). A total of 18 USPs were identified in *S. mansoni*. The number of USPs annotated for *H. sapien*s (~50) is much higher possibly due to the differences in complexity between these organisms (Table I). In addition, the *S. mansoni* orthologue proteins were compared to eukaryotic protein-coding genes, generated from the GENES database in KEGG, confirming their identification. Structures of the UCH domains differed comparing fifteen subclades, and their length varied from 300 to 700 amino acids among orthologs. We report that this parasite possesses 12 out of 18 USP genes with complete UCH domains. This difference in UCH domains may indicate diversity in substrate specificity as described for other organisms [34], the presence of non-functional USP or incomplete sequences deposited on parasite databases. The *S. mansoni* USP proteins exhibiting a complete UCH domain displayed the well-conserved Cys and His boxes, which include the catalytic triad formed by Cys, His and Asp residues (Fig. 1). It is reported that USPs constitute the largest DUB sub-family with approximately 56 members in humans and 16 in yeast. Such diversity highlights their involvement in diverse cellular processes [35].

**Table I:** Putative ubiquitin-specific proteases in *S. mansoni*

USP	*S. mansoni* putative orthologs	*KEGG Orthology Entry (KO)*	*KEGG Orthology name*	Length (aa)	Domain and Pfam number	Orthologue length (aa) in *Homo sapiens*	Pfam e-value
USP2	Smp_212390	K11833	USP2_21	563	UCH (PF00443) (171)	605	3.1e-66
USP5	Smp_069960	K11836	USP5_13, UBP14	916	zf-UBP	855	2.6e-16 1.4e-40
					UCH (341) UBA		1.6e-08
USP7	Smp_089180	K11838	USP7, UBP15	1412	MATH	1102	3.1e-12
					UCH (257)		7.4e-52
USP8	Smp_152000	K11839	USP8, UBP5	1027	IPPT	1118	1.7e-16
					UCH (685)		6.5e-69
USP9x	Smp_153690	K11840	USP9_24	811	UCH	918	9.7e-22
USP10	Smp_005280	K11841	USP10, UBP3	597	UCH (91)	802	5.9e-31
USP14	Smp_084740	K11843	USP14, UBP6	178	UCH (52)	494	3.9e-42
USP15	Smp_128770	K11835	USP4_11_15,	945	DUSP	951	3.8e-14
			UBP12		UCH (296)		3.4e-78
USP16	Smp_074200.1	K11844	USP16_45	622	UCH (44)	823	4.9e-30
USP20	Smp_021300	K11848	USP20_33	881	UCH (34)	914	6.1e-66
					DUSP		1.5e-12
USP22	Smp_074400	K11366	USP22_27_51, UBP8	489	UCH (181)	525	3.5e-52
USP24	Smp_198740	K11840	USP9_24	2235	UCH (229)	2620	4e-17
USP30	Smp_122960.1	K11851	USP30	595	UCH (58)	517	1.4e-21
USP36-42	Smp_046430	K11855	USP36_42	799	UCH (178)	1123	2e-49
USP39	Smp_017890	K12847	USP39, SAD1	577	zf-UBP	565	1.5e-12
					UCH		7.5e-37
USP46	Smp_000710	K11842	USP12_46	414	UCH (47)	366	8e-67
USP48	Smp_196290	K11858	USP48	1281	UCH (95)	1035	2.3e-12
USP49-44	Smp_123630	K11834	USP44_49	823	zf-UBP	640	2.7e-18
					UCH (201)		1.1e-63

All enzymes are classified as ubiquitin thioesterase

**Fig. 2:**
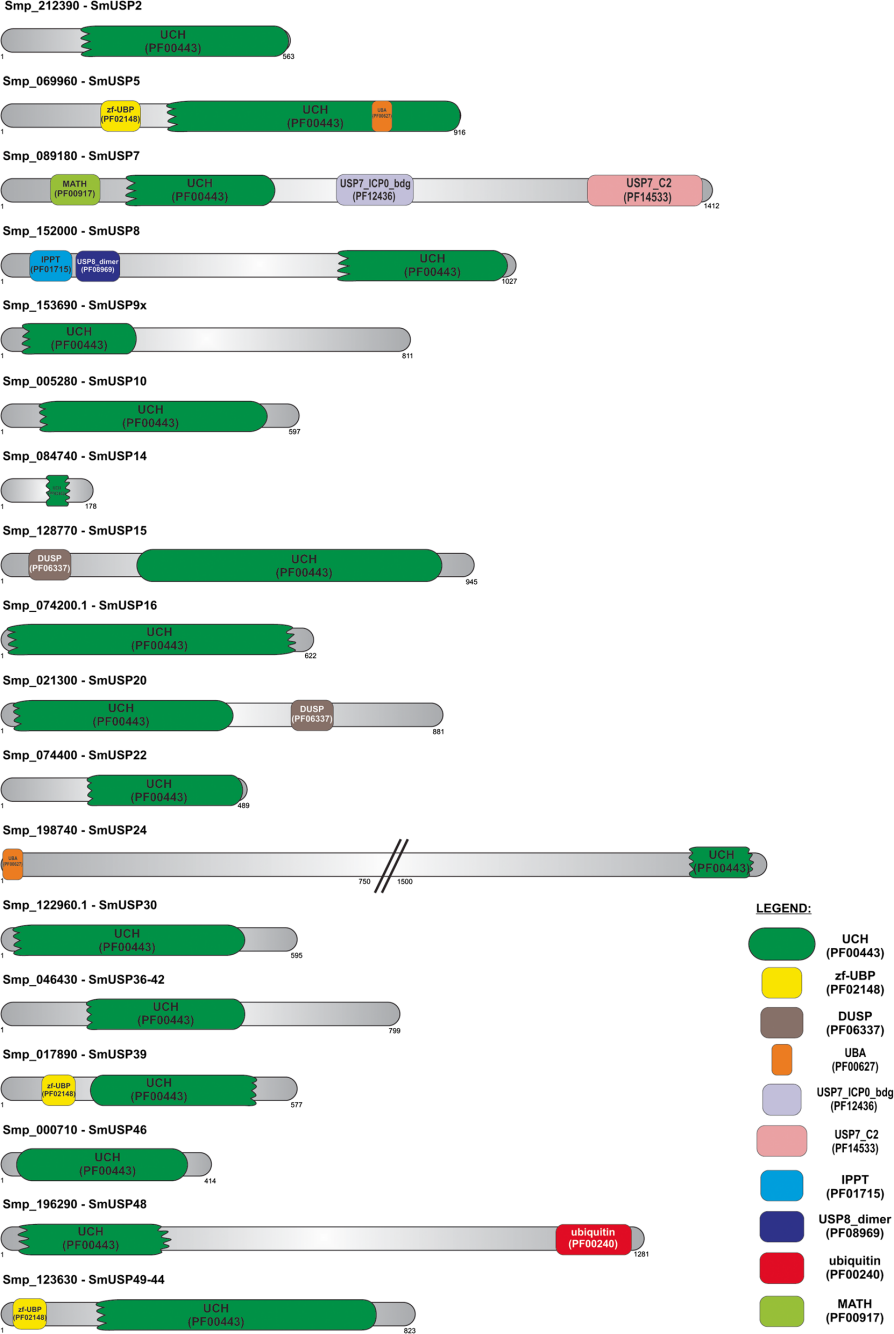
Schematic diagram of conserved protein domains in *S. mansoni* USPs A comparative analysis of the domains in *Sm*USP2, *Sm*USP5, *Sm*USP7, *Sm*USP8, *Sm*USP9×, *Sm*USP10, *Sm*USP14, *Sm*USP15, *Sm*USP16, *Sm*USP20, *Sm*USP22, *Sm*USP24, *Sm*USP30, *Sm*USP36-42, *Sm*USP39, *Sm*USP46, *Sm*USP48 and *Sm*USP49-44

In addition, conserved domains present in each USP were investigated (Fig. 2). USPs contain a diverse range of ancillary domains whose roles are poorly characterized for the majority of catalogued and annotated USPs [35]. This characteristic has been conserved for *S. mansoni* USPs. *Sm*USP2, *Sm*USP9x, *Sm*USP10, *Sm*USP14, *Sm*USP16, *Sm*USP22, *Sm*USP30, *Sm*USP36-42 and *Sm*USP46 contain only the UCH domain (PF00443). *Sm*USP5, *Sm*USP39 and *Sm*USP49-44 exhibit the zf-UBP domain (PF02148), which is a relatively small motif responsible for docking interactions with their target molecules [36]. Furthermore, *Sm*USP5 and *Sm*USP24 possess the UBA (ubiquitin associated) domain (PF00627) found in diverse proteins involved in the ubiquitin/proteasome pathway, DNA excision-repair, and cell signalling via protein kinases [37]. *Sm*USP7 contains three unique domains: MATH (Meprin and TRAF-Homology) (PF00917), USP7_ICPO (PF12436) and USP7_C2 (PF14533). The MATH domain, which participates in protein-protein interactions, is also found in intracellular TRAFs (tumour necrosis receptor-associated factors) and extracellular meprins [38]. USP7_ICP0, found only in *Sm*USP7, is known to interact with the herpesvirus 1 trans-acting transcriptional protein ICP0/VMW110 [39]. The third domain, USP7_C2, is found at the C-terminus of USP7, and its function is unclear. Only *Sm*USP8 contains the IPPT domain (PF01715) related to ATP binding and the USP8_dimer (PF08969) found at its N-terminus and likely involved in homodimer formation [40]. *Sm*USP15 and *Sm*USP20 also contain the DUSP (domain present in ubiquitin-specific protease) (PF06337), located at the N- and C-terminal sides of their UCH domains, respectively. The function of DUSP is unknown; however, it may play a role in protein/protein interaction or substrate recognition [35]. *Sm*USP48 is unique among the investigated molecules as it contains the ubiquitin domain (PF00240) at its C-terminus. Understanding the interaction of these protein domains provides insights into the diverse biological activities exhibited by *Sm*USP enzymes. Furthermore, the presence of the DUSP domain is not indicative of catalytic activity. A recent report showed that Usp39 lacks catalytic activity *in vitro* and is unable to cleave ubiquitin from ubiquitylated Aurora B *in vivo* [41].

**Fig. 3:**
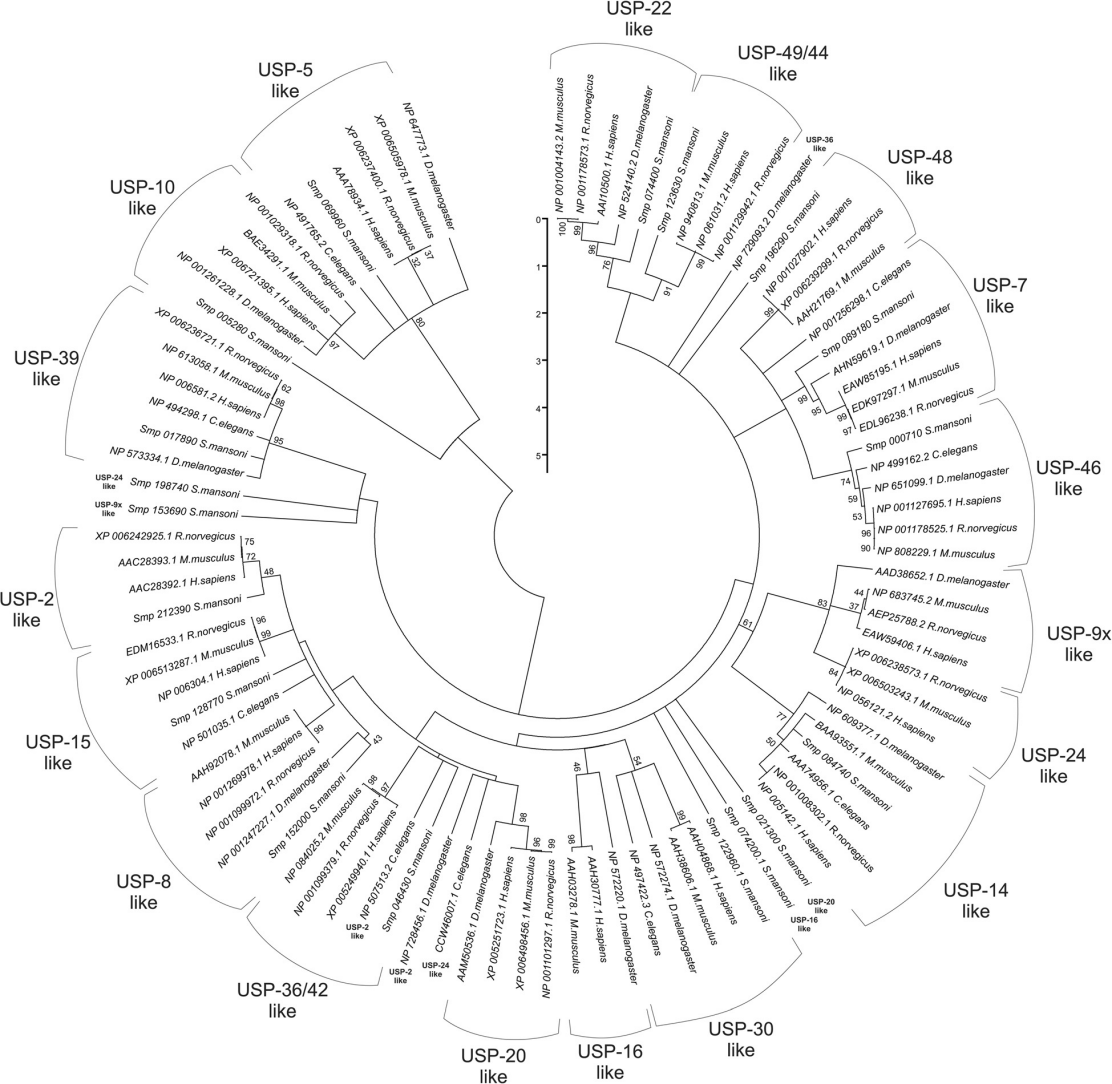
Phylogenetic tree of *S. mansoni* USP Conservation of *Sm*USP2, *Sm*USP5, *Sm*USP7, *Sm*USP8, *Sm*USP9x, *Sm*USP10, *Sm*USP14, *Sm*USP15, *Sm*USP16, *Sm*USP20, *Sm*USP22, *Sm*USP24, *Sm*USP30, *Sm*USP36-42, *Sm*USP39, *Sm*USP46, *Sm*USP48 and *Sm*USP49-44. Multiple alignments were performed using Mega 5.0 with bootstrap analysis. Branches corresponding to partitions reproduced in less than 50 % of the bootstrap replicates are collapsed [57]. The percentage of replicate trees in which the associated taxa clustered together in the bootstrap test (1000 replicates) is shown next to the branches. The tree was drawn to scale, with branch lengths representing the evolutionary distances used to infer the phylogenetic tree

A phylogenetic tree generated with the neighbour-joining method was used to separate the putative *Sm*USPs from their respective orthologues in *H. sapiens*, *M. musculus*, *R. norvegicus*, *C. elegans* and *D. melanogaster* (Fig. 3). Most of the *S. mansoni* USP proteins clustered with their orthologues into 15 subclades: USPs 2, 5, 7, 8, 10, 14, 15, 22, 30, 36–42, 39, 46, 48 and 49–44. The putative USP proteins 9x, 16 and 24 did not cluster in the same clade as their orthologues due to the incompleteness of their UCH domains. One exception was *Sm*USP20s, which exhibits the complete domain but did not cluster with their orthologs.

### Distinct expression of *Sm*USPs is observed during *S. mansoni* development

**Fig. 4:**
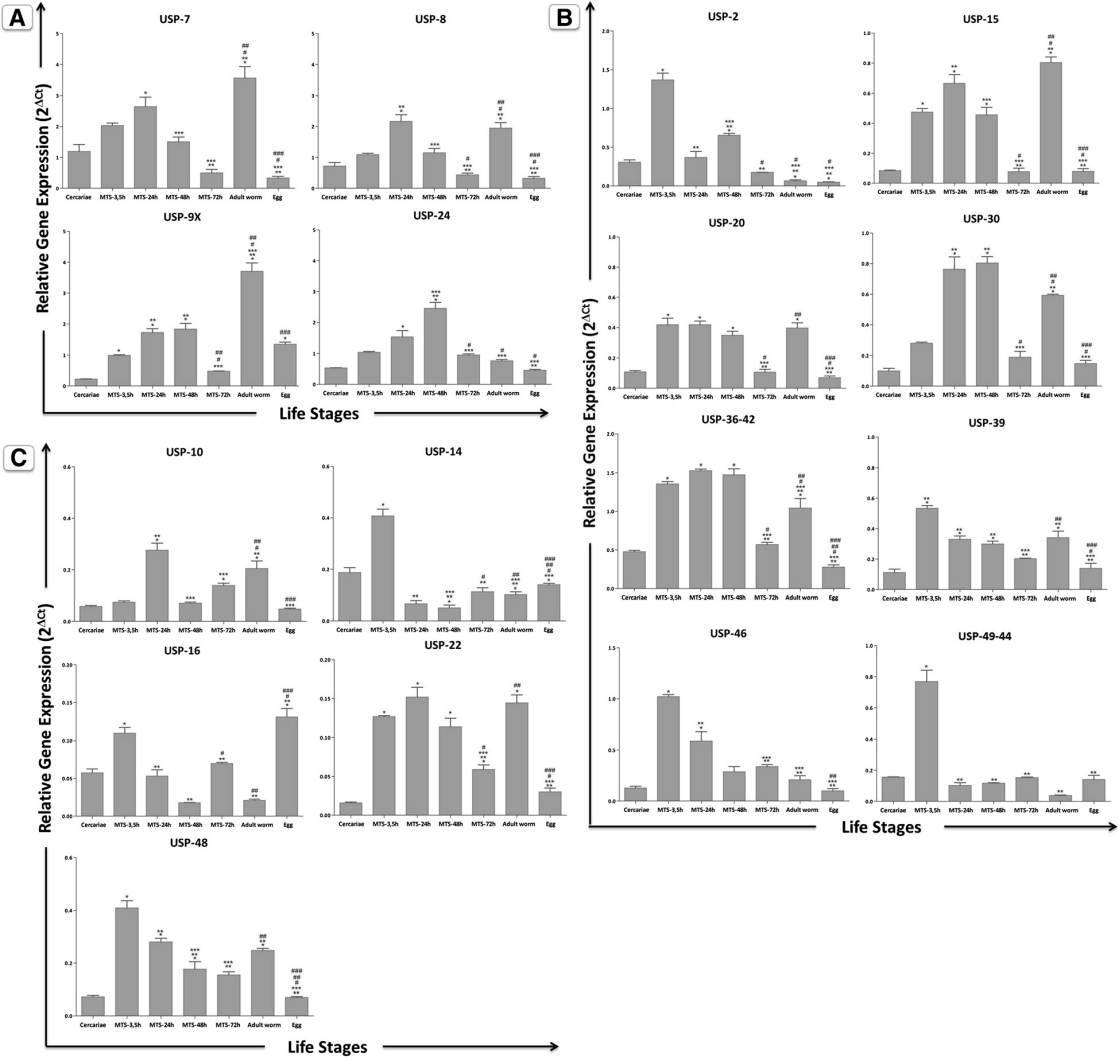
Differential expression of USP genes in various developmental stages of *S. mansoni* The mRNA expression levels of *Sm*USPs were measured, using three replicates, in the following stages: cercariae, MTS-3.5 h, MTS-24 h, MTS-48 h, MTS-72 h, adult worms and eggs using quantitative RT-PCR. Expression levels were calibrated according to the comparative 2^−ΔCt^ method using the constitutively expressed *Sm*EIF4E as an endogenous control (ANOVA followed by Tukey’s pairwise comparison p < 0.05). *: different from cercariae; **: different from MTS-3.5 h; ***: different from MTS-24 h; ^#^: different from MTS-48 h; ^##^: different from MTS-72 h; ^###^: different from adult worms. **a**) *Sm*USP7, *Sm*USP8, *Sm*USP9x and *Sm*USP24. **b**) *Sm*USP2, *Sm*USP15, *Sm*USP20, *Sm*USP30, *Sm*USP36-42, *Sm*USP39, *Sm*USP46 and *Sm*USP49-44. **c**) *Sm*USP10, *Sm*USP14, *Sm*USP16, *Sm*USP22 and *Sm*USP48

*Sm*USP transcript levels were analysed by qRT-PCR at different developmental stages of *S. mansoni:* cercariae, MTS-3.5 h, MTS-24 h, MTS-48 h, MTS-72 h, paired adult worms and eggs. Three biological and technical replicates were performed. The putative *Sm*USP transcripts analysed were detected in all investigated stages (Fig. 4). A 2-fold change in the expression of USP genes, relative to EIF4E transcript levels, was considered of biological relevance [42, 43]. According to their expression profile, *Sm*USPs were divided into three groups, to account for their high (0.5-5), medium (0.2-1.5) and low expression (0.02-0.5) levels. The *Sm*USPs exhibiting high expression profiles were *Sm*USP7, *Sm*USP8, *Sm*USP9x and *Sm*USP24 (Fig. 4a). Those showing medium expression were *Sm*USP2, *Sm*USP15, *Sm*USP20, *Sm*USP30, *Sm*USP36-42, *Sm*USP39, *Sm*USP46 and *Sm*USP49-44 (Fig. 4b). The *Sm*USPs displaying low expression profiles were *Sm*USP10, *Sm*USP14, *Sm*USP16, *Sm*USP22 and *Sm*USP48 (Fig. 4c).

The expression profile of *Smusp5* has been previously described [44]. Of the 17 analysed transcripts, none of the USPs was up-regulated in the cercariae and MTS-72 h stages (USP2, USP5, USP7, USP8, USP9x, USP10, USP14, USP15, USP16, USP20, USP22, USP24, USP30, USP36-42, USP39, USP46, USP48, USP49-44). In contrast, *Smusp2*, *Smusp39*, *Smusp46* and *Smusp49-44* were up-regulated in early schistosomula (*p* < 0.05). Extending the culture period to 24 h, *Smusp8* transcripts were observed at increased levels (*p* < 0.05). Concerning MTS-48 h, *Smusp24* was up-regulated in this stage (*p* < 0.05). *Smusp7*, *Smusp9x* and *Smusp15* transcripts presented at high levels in adult worms (*p* < 0.05). *Smusp20* was up-regulated in three stages: MTS-3.5 h, MTS-48 h and adult worms (*p* < 0.05). *Smusp30* was also up-regulated in three stages: MTS-24 h, MTS-48 h and adult worms (*p* < 0.05). *Smusp36-42* was up-regulated in early schistosomula (MTS-3.5 h MTS-24 h and MTS-48 h) (*p* < 0.05). Furthermore, *Smusp7*, *Smusp8*, *Smusp9x* and *Smusp24* were more highly expressed USPs in *S. mansoni*. Overall, upon examining the expression levels of all USP genes, it was found that the *Sm*USPs were more abundant in schistosomula and adult worms when compared with cercariae and eggs. This finding corroborates with previous data from our group, which demonstrated low levels of free, non-conjugated ubiquitin in the cercariae stage followed by its increase during schistosomula development up to adult worms [44]. In parallel, western blot analysis revealed the accumulation of ubiquitinated proteins in cercariae and schistosomula cultured up to 5 days relative to other intra-mammalian stages, suggesting that *Sm*USPs are less active at this stage [44]. This also coincides with the reported lower proteasomal proteolysis observed during early schistosomula compared with that found for the adult worm stage [17]. Whether the accumulation of ubiquitinated proteins are due to low levels of USPs transcripts, decreased proteasomal activity or their combined effects remains to be elucidated. These data reinforce the hypothesis that a major function of USP members in *S. mansoni* is the regulation of protein stability during the cercariae to adult worm development. The high levels of ubiquitinated conjugates found in early schistosomula likely indicates ubiquitination linked to alternative protein fates, such as sub-cellular localization and lysosome mediated degradation [45–47]. The importance of the ubiquitin cycle to protein stability is now recognized in all eukaryotic cells as a key mechanism for maintenance of cell viability [48].

The cellular roles of DUBs are as wide as that of the ubiquitin-proteasome system itself given the involvement of the Ub system in intracellular signalling [7]. Here, we selected some examples to illustrate their broader functional categorization. The proteasome has both ubiquitin ligases and DUBs that associate with it and several DUB-ligase pairs interact directly, including BRCC36-BRCA1, BAP1-BRCA1, USP4-Ro52, USP7-MDM2, USP8-GRAIL, USP20-pVHL, USP33-pVHL and USP44-APC [49]. Although the E3 repertoire in *S. mansoni* is not known, previous analyses from our group suggest the conservation of the MDM2, GRAIL and APC in the parasite genome (data not shown), indicating a role for USP49-44 in cell cycle progression. USP7 removes ubiquitin not only from p53 itself but also from the p53 E3 ubiquitin-ligase MDM2. These combined effects determine functional p53 levels, creating an important role for USP7 in p53-dependent stress responses. Our group showed that the p53 orthologues, p63 and p73 [50], are up-regulated in MTS-3.5 h and MTS-5 days [51]. We also observed a differential expression profile for *Smusp7. Smusp8* and *Smusp9x* were highly expressed throughout the parasite cycle. USP8 and USP9 appear to be involved in the control of mammalian cell proliferation, inducing apoptosis [52], indicating a role of these enzymes in parasite remodelling. *Smusp14* transcript level is higher in cercariae when compared with adult worms, suggesting that *Sm*USP14 can inhibit proteasome function noncatalytically, as previously observed for its yeast orthologue Ubp6 [53]. These data could explain, at least in part, the low rate of proteasome-dependent proteolysis detected in cercariae when compared with adult worms [17]. *Smusp20* expression is up-regulated in intra-mammalian stages, particularly in MTS-3.5 h. Orthologues of USP20 seem to be involved in hypoxia signalling, and the possibility of this protease to regulate the schistosomula development will be investigated [54]. Additionally, *Smusp24* and *Smusp30* were three times more abundant in MTS-48 h when compared with adult worms. Considering that USP24 was recently identified as a novel regulator of DDB2 (damage-specific DNA-binding protein 2) stability [55] and the implication of USP30 in the maintenance of mitochondrial morphology [56], *Sm*USP24 and *Sm*USP30 could be necessary during parasite remodelling. Furthermore, the transcription levels of *Smusp36-42* are similar when comparing MTS-3.5 h, 24 and 48 h, reinforcing the hypothesis that specific *Sm*USPs can be important for early schistosomula development.

## Conclusions

In conclusion, we describe the USP enzyme repertoire in *S. mansoni* and their regulated expression in the parasite life cycle. This differential profile of transcripts can reflect the stage-specific subset of their target ubiquitinated substrates during the parasite’s life cycle. Our results raise a number of interesting questions concerning the regulation of *Sm*USP activities and their role during schistosome development. USPs are temporally and spatially controlled and most often act as part of multi-protein complexes [56, 55]. Moreover, a single USP can act upon various substrates, and its activity can be regulated by several DUBs. Further experiments shall clarify the functions of putative *Sm*USPs in *S. mansoni.*

## Additional file

Additional file 1: Table S1. Sequence accession numbers and primer pairs. **Table S2.** Putative ubiquitin-specific proteases in *S. mansoni* and *S. japonicum.*
