# Supplementary material for: Ubiquitin-specific proteases are differentially expressed throughout the Schistosoma mansoni life cycle
Source: Parasit Vectors. 2015 Jun 26;8:349. doi: 10.1186/s13071-015-0957-4 (PMC4485857; doi:10.1186/s13071-015-0957-4)
Supplement: Additional file 1: Table S1. — Sequence accession numbers and primer pairs. Table S2. Putative ubiquitin-specific proteases in S. mansoni and S. japonicum. [file 13071_2015_957_MOESM1_ESM.docx]

**TABLE SI.** Sequence accession numbers and primer pairs

| Gene | Accession Numbers | Primers |
| --- | --- | --- |
| USP2 | Smp_212390 | F: 5’CACTCATATCACAGAGGTGG3’  R: 5’TCGGTAAGTGGAGCAGTG3’ |
| USP7 | Smp_089180 | F: 5’TTATGTCTCACCCGTCTC3’  R: 5’CCCACTCTCTACAGAACAG3’ |
| USP8 | Smp_152000 | F: 5’GGCTGATTTGTTGGCTTC3’  R: 5’TGGTTTAGGTGCTAGTGG3’ |
| USP9x | Smp_153690 | F: 5’CAATCAACCGCCTGCTTG3’  R: 5’TGCTGGTTCGGACTCCATAC3’ |
| USP10 | Smp_005280 | F: 5’GCACACCGTATATTCTTCTC3’  R: 5’GCCAACATACACATACACAC3’ |
| USP14 | Smp_084740 | F: 5’CAATCCCTCAAGCCAAAC3’  R: 5’AAGTTCGTAGTACCCAGAG3’ |
| USP15 | Smp_128770 | F: 5’TAGAACAAGAGCGTCCAC3’  R: 5’GCAATCAGGGAGGCATAC3’ |
| USP16 | Smp_074200.1 | F: 5’TAGTCCTGGGATGTTTCG3’  R: 5’AGCAGTCCAATAGAGCAC3’ |
| USP20 | Smp_021300 | F: 5’TCTGCCCTGAAAGACATC3’  R: 5’CACGAGGAACTGAAACAC3’ |
| USP22 | Smp_074400 | F: 5’CTGTAAATGGCTGCTCTG3’  R: 5’ACGGGTGTATGGGTCAAC3’ |
| USP24 | Smp_198740 | F: 5’TGGTGTTCATTGGGATGTCC3’  R: 5’CATGTTGCACCACCATTACG3’ |
| USP30 | Smp_122960.1 | F: 5’CGACGAGCAAACGAGGAAG3’  R: 5’GCTGCCATTGCTTGCATC3’ |
| USP36-42 | Smp_046430 | F: 5’GACCAATGTCGTCTACTGC3’  R: 5’GACCCATCAGAACCACAATC3’ |
| USP39 | Smp_017890 | F: 5’CCTCACACTCATCGTTCTC3’  R: 5’ATTCAGGTTTGGGCTGTG3’ |
| USP46 | Smp_000710 | F: 5’TCGGAGTAGATGCTGAAG3’  R: 5’GGCACCTAGTTTCATTGG3’ |
| USP48 | Smp_196290 | F: 5’AGGCATTGTTGATCCAGGAG3’  R: 5’TACACCAACGTGCTGGAAAC3’ |
| USP49-44 | Smp_123630 | F: 5’GGATTGGTGTGTTGTTCTTC3’  R: 5’CGCATCTCCATCTTGTAG3’ |

**TABLE SII.** Putative ubiquitin-specific proteases in *S. mansoni* and *S. japonicum*

| **USP** | ***S. mansoni* putative**  **orthologues** | **Length (aa)** | ***S. japonicum* putative**  **orthologues in GeneDB** | **Length (aa)** | ***S. japonicum* putative**  **Orthologs**  **in NCBI** | **Length (aa)** | **Pfam**  **e-value**  ***S. japonicum*** | **Sequence similarity (%)**  **in GeneDB** |
| --- | --- | --- | --- | --- | --- | --- | --- | --- |
| USP2 | Smp_212390 | 563 | Sjp_0079320.1 | 270 | CAX73508.1 | 496 | 1.9e-12 | 82 |
| USP5 | Smp_069960 | 916 | Sjp_0047560.1 | 767 | CAX72904.1 | 916 | 6e-26 | 62 |
| USP7 | Smp_089180 | 1412 | Sjp_0030710.1 | 881 | AAW27852.1 | 506 | 4.8e-26 | 91 |
| USP8 | Smp_152000 | 1027 | Sjp_0050280.1 | 246 | AAX27807.2 | 222 | - | 59 |
| USP9x | Smp_153690 | 811 | Sjp_0032170.1 | 2905 | AAX25064.2 | 180 | 3.2e-33 | 65 |
| USP10 | Smp_005280 | 597 | Sjp_0037330.1 | 572 | AAX27683.2 | 300 | 1.1e-32 | 80 |
| USP14 | Smp_084740 | 178 | - | - | - | - | - | - |
| USP15 | Smp_128770 | 945 | Sjp_0070290.1 | 882 | CAX74618.1 | 933 | 4.3e-78 | 82 |
| USP16 | Smp_074200.1 | 622 | Sjp_0061650.1 | 742 | CAX83082.1 | 148 | 3.4e-48 | 75 |
| USP20 | Smp_021300 | 881 | Sjp_0010020.1 | 321 | - | - | - | 66 |
| USP22 | Smp_074400 | 489 | Sjp_0023310.1 | 458 | AAX26655.2 | 174 | 1.6e-24 | 87 |
| USP24 | Smp_198740 | 2235 | Sjp_0079300.1 | 1494 | - | - | 8.4e-22 | 71 |
| USP30 | Smp_122960.1 | 595 | Sjp_0006010.1 | 531 | AAW24643.1 | 224 | 2.7e-16 | 79 |
| USP36-42 | Smp_046430 | 799 | Sjp_0054130.1 | 1719 | AAW27576.1 | 233 | 5.7e-17 | 54 |
| USP39 | Smp_017890 | 577 | Sjp_0083630.1 | 332 | AAW27682.1 | 250 | 2.2e-20 | 94 |
| USP46 | Smp_000710 | 414 | Sjp_0106640.1 | 266 | AAW27333.1 | 412 | 2.7e-28 | 94 |
| USP48 | Smp_196290 | 1281 | Sjp_0034090.1 | 994 | AAW26304.1 | 666 | 9.9e-15 | 83 |
| USP49-44 | Smp_123630 | 823 | Sjp_0104920.1 | 845 | AAX27717.2 | 235 | 4.9e-63 | 85 |
